# Retrograde thoracic duct embolization for adult-onset plastic bronchitis with idiopathic lymphatic flow anomaly

**DOI:** 10.1016/j.radcr.2025.06.068

**Published:** 2025-07-18

**Authors:** Hayden L. Hofmann, Jesse Han, Jenanan Vairavamurthy, Ramon R. Ter-Oganesyan

**Affiliations:** a Department of Radiology, Division of Interventional Radiology, Keck School of Medicine, University of Southern California, Los Angeles, CA, USA; b Department of Radiology, Division of Interventional Radiology, Icahn School of Medicine at Mount Sinai, New York, NY, USA; c Department of Radiology, Division of Interventional Radiology, Los Angeles General Medical Center, Los Angeles, CA, USA

**Keywords:** Plastic bronchitis, Thoracic duct embolization, Lymphatic malformations

## Abstract

We report a case of a patient who developed adult-onset plastic bronchitis (PB) due to aberrant lymphatic anatomy, which resulted in direct lymph drainage into the left lower lobe bronchus. After failure of medical management, successful thoracic duct embolization was performed targeting the aberrant lymphatic duct via transnodal lymphangiography and a retrograde transvenous lymphatic access. Following intervention, the patient reported no adverse events and complete resolution of symptoms without any chronic cough or cast expectoration. The case supports the advantages of transcatheter lymphatic embolotherapy including its minimally invasive nature and short recovery period and hospital stay. Additionally, the case provides evidence for the use of transcatheter thoracic duct n-butyl cyanoacrylate (n-BCA) embolization for managing lymphatic malformations in patients presenting with plastic bronchitis.

## Introduction

Plastic bronchitis ac(PB) is a rare respiratory condition characterized by the formation of branching bronchial casts, composed of lipid-laden macrophages and neutrophil mucus plugs, which can cause life-threatening asphyxiation. PB is more commonly observed in the pediatric population, often following surgical interventions for congenital heart diseases [1]. In adults, only a few cases of PB have been described, mostly associated with congenital anomalies of the pulmonary lymphatic anatomy resulting in direct drainage into the bronchial tree [2,3]. For patients with PB that fail medical therapy, interventions such as transcatheter embolization or surgical ligation of the thoracic duct (TD) have shown improvement or resolution of symptoms. We present a case of a patient with adult onset PB secondary to likely acquired lymphatic malformation, resulting in direct lymph drainage into the left lower lobe bronchus.

## Case report

**Fig. 1:**
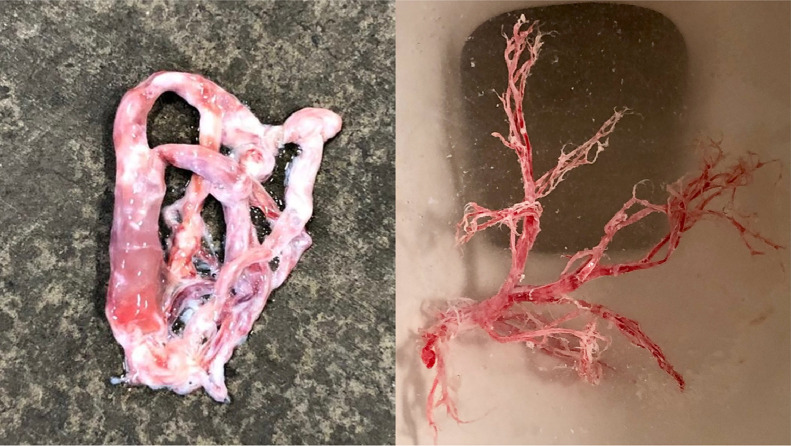
Pictorial representation of the expectorated bronchial casts.

Approval from the institutional review board was not required for this case report. The case involves a 31-year-old male with history of expectoration of bronchial casts for approximately four years (Fig. 1), who presented with acute shortness of breath and hemoptysis. After bronchoscopy and two lymphangiography procedures at an outside institution, he was diagnosed with adult-onset plastic bronchitis (PB). The previous lymphangiograms demonstrated anomalous lymphatic drainage in the left lung via an aberrant lymphatic duct distinct from the TD. No intervention was performed on either occasion as percutaneous TD access could not be obtained. Despite medical management, the patient continued to experience recurrent cast expectoration, and additionally developed hemoptysis, hypoxia, segmental pulmonary embolism, and pericardial effusion, necessitating additional hospitalizations. He was referred to our interventional radiology service for an additional attempt at lymphangiography and thoracic duct embolization.

**Fig. 2:**
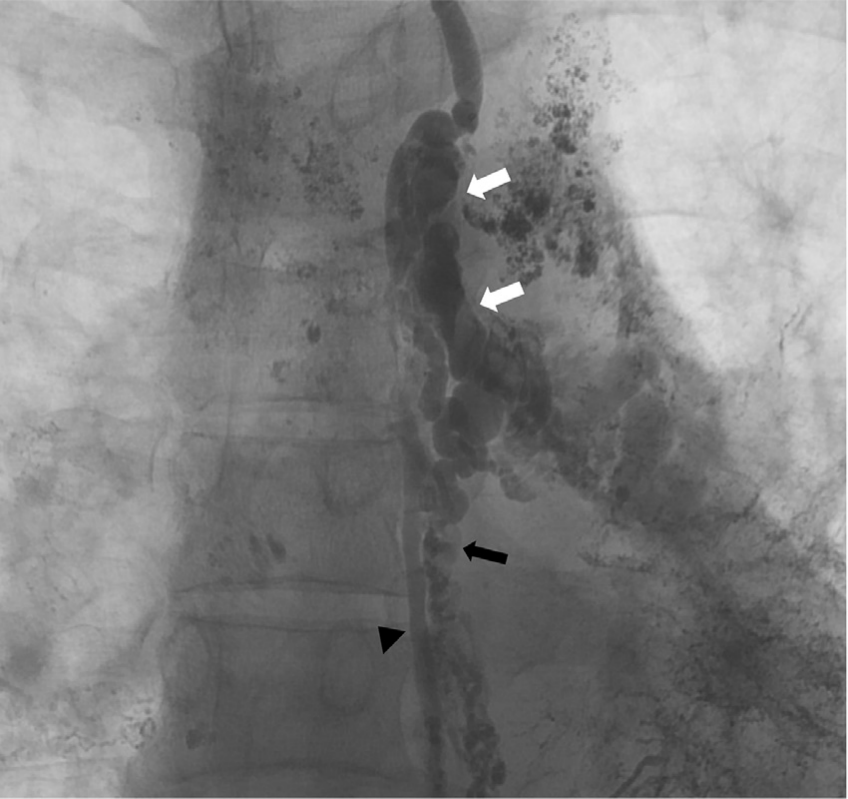
Lymphangiogram performed using water-soluble contrast with transabdominal microcatheter in the TD. The image demonstrates opacification of large distinct tortuous aberrant lymphatic duct (white arrows) branching off the upper portion of the TD (black arrowhead), coursing towards the left lower lobe bronchial tree in parallel to the TD. Additional smaller lymphatic channels were seen with small collaterals connecting to the TD more caudally (black arrow).

Under moderate sedation, bilateral trans-nodal lymphangiography was performed with Lipiodol (Guerbet, Villepinte, France) ethiodized oil via the groin lymph nodes using ultrasound and fluoroscopic guidance. Following opacification of the TD, percutaneous transperitoneal cysterna chyli access was achieved under fluoroscopic guidance using a 21-gauge Chiba needle (Cook Medical, Bloomington, Indiana), and a 0.018-inch Nitrex guidewire (EV3, North Plymouth, Minnesota) was passed into the TD. Subsequently a 2.7-Fr Progreat microcatheter (Terumo Medical, Tokyo, Japan) was advanced into the TD, and lymphangiography was performed using water-soluble contrast. The lymphangiogram revealed a patent thoracic duct with normal drainage into the central left subclavian vein. Additionally, a distinct large, tortuous aberrant lymphatic duct branching off the upper portion of the TD was observed, coursing caudally toward the left lower lobe bronchial tree in parallel to the TD. Multiple other lymphatic channels were seen running parallel to the TD, all connecting to the aberrant duct centrally (Fig. 2). Small collateral connections to the caudal portion of the TD were also observed.

Multiple attempts to access the aberrant duct with the transperitoneal microcatheter were unsuccessful given the sharp downward angulation of the duct. Exchange was then made to a SwiftNinja microcatheter (Merit Medical Systems, South Jordan, Utah) and additional attempts to access the duct utilizing the torquing ability of the microcatheter were unsuccessful. Retrograde transvenous TD access was then pursued. The left basilic vein was accessed with ultrasound guidance, a 6-Fr side-arm vascular sheath was placed, followed by the insertion of a 5-Fr angled catheter. Using a Fathom guidewire (Boston Scientific, Marlborough, Massachusetts) the transperitoneal SwiftNinja microcatheter was manipulated through the TD into the left subclavian vein. A 300 cm 0.018-inch Nitrex guidewire was then inserted through the SwiftNinja catheter and captured using a 10-mm Amplatz Goose Neck snare (EV3, North Plymouth, Minnesota) through the basilic vein access. The snare and wire were then pulled out of the venous access sheath for through-and-through access. Over the wire, the microcatheter was retracted into the TD and a 5-Fr angled catheter was advanced retrogradely into the central TD. A Progreat 2.7 microcatheter was then inserted coaxially to select the aberrant duct coursing toward the left lower lobe bronchus. The microcatheter was placed peripherally and additional lymphangiography was performed, including a cone-beam CT, confirming an extensive network of lymphatic channels surrounding the left lower lobe bronchus, which coalesced centrally, connecting to the TD.

**Fig. 3:**
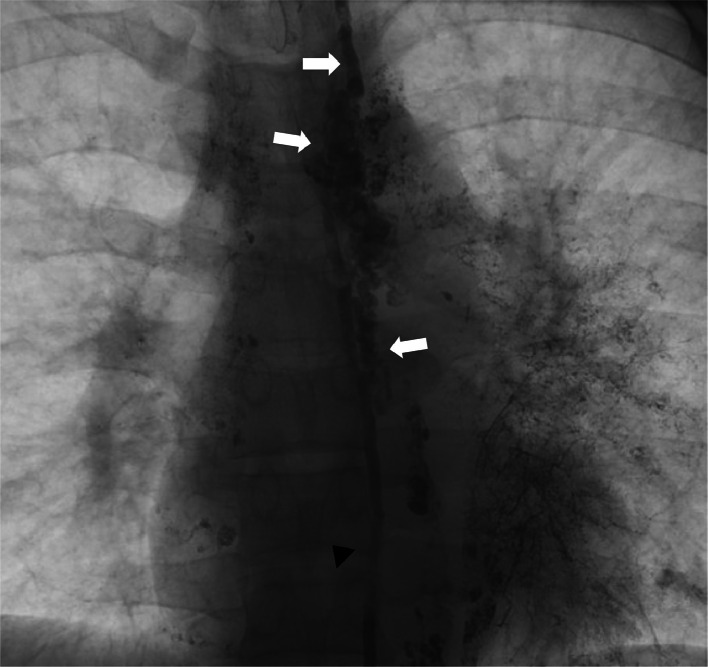
Lymphangiogram performed following embolization of the aberrant lymphatic channel demonstrates n-BCA glue in the aberrant channel (white arrows) and the thoracic duct (black arrowhead).

Via the microcatheter, the anomalous lymphatic channel was embolized using TRUFILL n-BCA liquid embolic (Cerenovus, Irvine, California), from the level of the left lower lobe bronchus up to the TD. Fluoroscopic monitoring illustrated the filling of the dominant and smaller anomalous lymph channels on the left side, as well as the mid-thoracic duct. Postembolization lymphangiography via the transabdominal access confirmed occlusion of the thoracic duct, without further opacification of the anomalous lymphatic channels (Fig. 3).

The patient was discharged home the same day following a standard recovery period, without any immediate complications. At one week follow-up, the patient reported no adverse events and complete resolution of symptoms without any chronic cough or cast expectoration. The results were durable at the three month follow-up.

## Discussion

Adult-onset plastic bronchitis is a rare condition, and its management with thoracic duct embolization remains poorly studied. However, prior literature has established similar technical approaches for embolization of the thoracic duct in treatment of other lymphatic conditions including chylous ascites and chylothorax. Specifically, the transvenous retrograde approach for accessing the thoracic duct is well-documented for embolization of chyle leaks [[4], [5], [6]]. Retrograde transvenous access can be a challenge due to poor visualization of the lesion on the initial inguinal intranodal lymphangiogram as well as poor visualization of the access into the thoracic duct from the subclavian venography due to the ostial thoracic duct valves. Additionally, the presence of valves and anatomical variability of the thoracic duct can further complicate navigation to the target lesion [5]. Percutaneous transabdominal access is often used as a first option in accessing the thoracic duct given retrograde transvenous access is often more technically challenging and can often have low technical success rates [7]. Both techniques were employed during this case given the patient’s complex anatomy and need for through-and-through access. This case highlights that, despite its technical challenges, retrograde transvenous access can be utilized in treatment of thoracic duct embolization for PB.

Thoracic duct embolization has also been reported in cases of plastic bronchitis, including those specifically involving adult patients [8,9]. In one case series, four of six patients who underwent thoracic duct embolization experienced complete resolution of symptoms immediately following the procedure. The remaining two patients required reintervention and ultimately had only partial symptom improvement [8]. In a separate case study, the patient who underwent lymphatic duct embolization achieved complete symptom resolution postoperatively [9]. The patient in this study experienced rapid and significant symptom improvement immediately following intervention and out to three months follow up. Furthermore, recent comparisons between selective lymphatic duct embolization (SLDE) and thoracic duct embolization for thoracic lymphatic flow disorders have shown that SLDE preserved thoracic duct patency while achieving comparable outcomes [10]. Given the complex anatomy of the aberrant lymphatic channels, the operators opted for thoracic duct embolization over SLDE for a more definitive and lasting solution for this patient who experienced significant morbidity requiring prior hospitalizations for hemoptysis, hypoxia, PE, and pericardial effusions. This case raises important questions about best practices in managing lymphatic flow anomalies given that prior studies primarily examine pediatric population. Further research is needed to assess the efficacy and long-term treatment outcomes for the smaller, more specific population of adult-onset plastic bronchitis.

## Conclusion

This case highlights the successful use of combined transabdominal and transvenous thoracic duct n-BCA embolization for managing lymphatic malformations in patients presenting with adult-onset plastic bronchitis.

## Consent for publication

Consent for publication was obtained for every individual person’s data included in the study.

## Patient consent

Informed consent for publication was obtain from the patient discussed in this case report.

## Appendix Supplementary materials

Supplemental Fig. 1: Pre intervention CT scan demonstrating lymphatic collection in the left lower lobe bronchus and parabronchial region. Image, image 1
