# Supplementary figures and images for: Retrograde thoracic duct embolization for adult-onset plastic bronchitis with idiopathic lymphatic flow anomaly
Source: Radiol Case Rep. 2025 Jul 18;20(10):4994–7. doi: 10.1016/j.radcr.2025.06.068 (PMC12301785; doi:10.1016/j.radcr.2025.06.068)

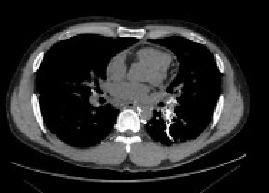

Supplement: Supplementary file 1 — Supplemental Fig. 1: Pre intervention CT scan demonstrating lymphatic collection in the left lower lobe bronchus and parabronchial region. [file mmc1.jpg]
